# Preoperative anaemia and thrombocytosis predict adverse prognosis in non‐metastatic renal cell carcinoma with tumour thrombus

**DOI:** 10.1186/s12894-021-00796-6

**Published:** 2021-02-27

**Authors:** Ruotao Xiao, Chuxiao Xu, Wei He, Lei Liu, Hongxian Zhang, Cheng Liu, Lulin Ma

**Affiliations:** 1 Peking University Health Science Centre, 100191 Beijing, People’s Republic of China; 2 Department of Urology, Peking University Third Hospital, No.49 North Garden Road, Haidian District, 100191 Beijing, People’s Republic of China

**Keywords:** Anaemia, Thrombocytosis, Renal cell carcinoma, Tumour thrombus, Prognosis

## Abstract

**Background:**

This study aimed to determine the prognostic value of preoperative blood parameters in renal cell carcinoma (RCC) and tumour thrombus (TT) patients that were surgically treated.

**Method:**

We retrospectively analysed clinicopathological data and blood parameters of 146 RCC and TT patients that were surgically treated. Univariate or multivariate Cox regression analyses were performed to determine the risk factors associated with progression-free survival (PFS) and overall survival (OS). Kaplan-Meier analysis and logistic regression were performed to study the risk factors. Receiver operating characteristic curves were applied to test improvements in the predictive accuracy of the established prognosis score.

**Results:**

On univariate and multivariate analysis, anaemia (HR 2.873, P = 0.008) and lymph node metastasis (HR 4.811, P = 0.015) were independent prognostic factors linked to OS. Besides, thrombocytosis (HR 2.324, P = 0.011), histologic subtype (HR 2.835, P = 0.004), nuclear grade (HR 2.069, P = 0.033), and lymph node metastasis (HR 5.739, P = 0.001) were independent prognostic factors associated with PFS. Kaplan–Meier curves revealed that patients with anaemia exhibited worse OS than those without it (P = 0.0033). Likewise, patients with thrombocytosis showed worse PFS than those without it (P < 0.0001). Adding the anaemia and thrombocytosis to the SSIGN score improved its predictive accuracy related to OS and PFS. Preoperative anaemia was linked to more symptom at presentation (OR 3.348, P = 0.006), longer surgical time (OR 1.005, P = 0.001), more blood loss (OR 1.000, P = 0.018), more transfusion (OR 2.734, P = 0.004), higher thrombus level (OR 4.750, P = 0.004) and higher nuclear grade (OR 3.449, P = 0.001) while thrombocytosis was associated with more symptom at presentation (OR 7.784, P = 0.007).

**Conclusions:**

Preoperative anaemia and thrombocytosis were adverse prognostic factors in non-metastatic RCC patients with TT. Also, both preoperative anaemia and thrombocytosis can be clinically used for risk stratification of non-metastatic RCC and TT patients.

## Background

Renal cell carcinoma (RCC) accounts for 3% of all malignant tumours worldwide [1]. With the advancement of surgical procedures and adjuvant therapy, the long-term prognostic outcome of RCC has become favourable with a 5-year survival rate of 80–90 % in most histological subtypes[2]. Based on pathological data, RCC subtype, tumour grade, sarcomatoid and rhabdoid features, and TNM stage are commonly used prognostic factors of RCC [3]. Combining these factors, several prognostic models have established to predict survival among the RCC patients and validated their usefulness externally[3].

Notably, preoperative blood parameters can be used to reflect the inflammatory status and health conditions of patients in an economic and convenient manner. The inflammation status has been well associated with genesis, progression, and metastasis of tumours [4]. Preoperative inflammation indicators such as neutrophils, lymphocytes, platelets, neutrophil-lymphocyte ratio (NLR), and the platelet lymphocyte ratio (PLR) have been reported for prognostic value in urologic malignancies such as prostate and bladder cancers [5, 6]. Previously, in RCC too, elevated neutrophils, platelets, NLR, PLR, and decreased lymphocytes were linked to poor survival rates [7–9]. Moreover, other preoperative blood parameters such as haemoglobin, AST/ALT, albumin have been suggested to hold prognostic values predicting survival in RCC [10]. Recently, a novel prognostic system incorporating preoperative blood parameters has also been designed to improve prognostic accuracy to predict metastatic RCC [11].

Intriguingly, 4–10 % of RCC patients exhibit thrombus either in the renal vein or inferior vena cava [12]. Due to the complexity of the inflammatory status and rarity of data, the prognostic significance of preoperative blood parameters has not been extensively studied yet. Therefore, this study was aimed to determine the prognostic value of preoperative blood parameters in RCC and TT patients that were surgically treated.

## Methods

### Patient population

**Fig. 1:**
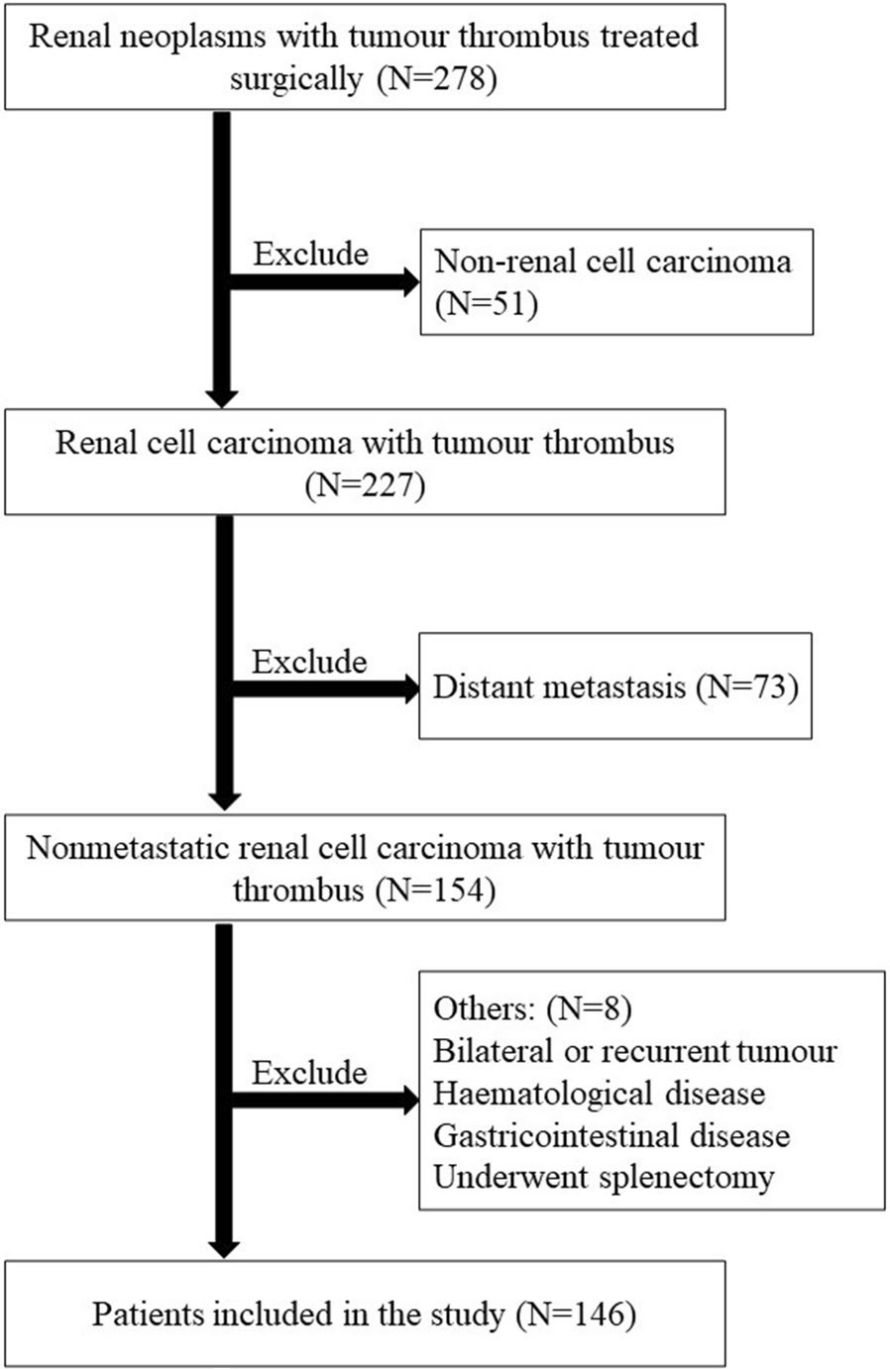
Flow chart showing the inclusion/exclusion criteria of the patients

After approval from the Peking University Third Hospital Medical Science Research Ethics Committee, we retrospectively analysed the clinicopathological data of 278 patients. These were diagnosed with renal neoplasms and tumour thrombus receiving nephrectomy and thrombectomy at our institute between Jan 2014 and Dec 2019. The excluding criteria were as follows: (1) non-renal cell carcinoma, (2) preoperative suspicious distant metastasis, (3) bilateral or recurrent tumour, (4) combined with hematological disease or gastrointestinal disease, (5) a past-history of splenectomy. The flow chart describing the inclusion criteria of patients is shown in Fig. 1. Finally, data belonging to 146 patients were used for further research. The corresponding clinicopathological data and blood parameters were obtained using the patients’ medical and pathological records.

### Clinical and pathologic evaluation

The clinicopathological variables included age, sex, body mass index (BMI), symptoms at presentation, American Society of Anesthesiologists (ASA) score, surgical data (approach, time, blood loss, segmental resection of vena cava, and transfusion), tumour thrombus level (TT), tumour size and side, and tumour pathology (histologic subtype, nuclear grade, lymph node status, necrosis, and invasion of venous wall). Tumour thrombus levels were defined according to the Mayo classification using the preoperative images [13]. Nuclear grade was defined according to Fuhrman grade before 2016 and WHO/IUSP classification after 2016 [14]. SSIGN score was used to stratify the study cohort in low-risk (score 2–4), intermediate-risk (score 5–7), and high-risk (score 8–11) groups [15]. Patients’ routine laboratory examinations were analysed for a period of one week before the surgery. The corresponding blood parameters including neutrophil count (NEU), platelet count (PLT), lymphocyte count (LYM), haemoglobin level (HGB), albumin level (ABL), serum creatine (SCR), plasma AST and ALT, serum calcium (SCa) and alkaline phosphatase (ALP) levels were obtained. NLR and PLR were calculated using the neutrophil and platelet counts. PLT ≥ 300 × 10^9^/L was defined as thrombocytosis. HGB ≤ 120 g/L for males and HGB ≤ 115 g/L for females were defined as anaemia.

### Follow up

After surgery, all patients were followed up and prognostic data were obtained through a clinic visit or by telephone until July 2020. Patients were recommended to a follow up for every 3 months in the first year, every 6 months in the next 2 years, and yearly thereafter. At each visit, laboratory examinations, X-ray, ultrasonic scan, or abdominal CT were performed. Patients who had recurrence or metastasis during follow-up were recommended to receive tyrosine kinase inhibitors (TKIs) as adjuvant therapy. Overall survival (OS) was calculated from the date of surgery to death and progression-free survival (PFS) was calculated from the date of surgery to tumour recurrence or metastasis.

### Statistical analysis

The prognostic value (OS and PFS) of preoperative blood parameters and clinicopathologic data were assessed using the univariate and multivariate Cox proportional hazards regression model. Potential predictors with a significance of P < 0.10 in the univariable Cox regression analysis or with clinic value were included in the subsequent multivariable regression analysis (with forward stepwise likelihood ratio method). The OS and PFS were estimated using the Kaplan–Meier curves and survival differences were compared using the log-rank test. Association among risk factors and clinicopathological characteristics was analysed using the logistic regression model. Time-dependent receiver operating characteristic (ROC) curves were performed to analyse the improvement of the modified model compared to the conventional model [16]. All statistical analyses were performed with SPSS Statistics 22.0 (IBM Corp, Armonk, NY, USA) and R software (Version 4.0.3). All comparisons were subjected to Two-tailed tests, and P < 0.05 was considered statistically significant.

## Results

The baseline clinical and pathological variables of 146 RCC and TT patients that were treated surgically are shown in Table 1. Among these, the majority (n = 113, 77.4 %) of patients were male. The patients’ median age was 60 year (IQR 54–66.25). Of these, the majority of patients had TT levels classified to Mayo 0–II (n = 118, 80.8 %) than Mayo III–IV (n = 28, 19.2 %). According to SSIGN score, patients in the low-risk (score2–4), intermediate-risk (score 5–7), and high-risk (score 8–11) groups were 49 (33.5 %), 76 (52.1 %), and 21 (13.4 %) respectively. At the last follow-up, 139 (95.2 %) patients were followed up for a median of 19 months (IQR 8–32). Twenty-eight (19.2 %) patients died with the mean OS of 46.82 months (median OS was not reached). The 1-year and 3-year cumulative OS rates were 90.3 % and 73.4 % respectively. Fifty-two (35.6 %) patients had disease progression with the median PFS of 37 months (IQR 22–60). The 1-year and 3-year cumulative PFS rates were 87.2 % and 50.7 % respectively.

**Table 1:** Univariate Cox regression analysis of prognostic factors correlated with overall survival and progression-free survival

Characteristics	All cohort (n = 146)
Age, y, median. (IQR)	60 (54–66.25)
*Sex, no. (%)*	
Male	113 (77.4)
Female	33 (22.6)
BMI, kg/m^2^, median. (IQR)	23.87 (21.44–26.65)
*Symptom at presentation, no. (%)*	
Absent	38 (26)
Present	108 (74)
*ASA score, no. (%)*	
I–II	129 (88.3)
III	17 (11.7)
*Surgical approach, no. (%)*	
Open	59 (40.4)
Laparoscopic	87 (59.6)
Surgical time, min, median. (IQR)	305.5 (224.25–400)
Blood loss, ml, median. (IQR)	600 (200–1650)
Transfusion, no. (%)	61 (41.8)
Segmental resection of vena cava, no. (%)	27 (18.5)
*Tumour side, no. (%)*	
Left	52 (35.6)
Right	94 (64.4)
Tumour size, cm, median. (IQR)	8.25 (6.48–10)
*Tumour thrombus level, no. (%)*	
0–II	118 (80.8)
III–IV	28 (19.2)
Invasion of venous wall, no. (%)	53 (41.4)
*Histology subtype, no. (%)*	
Clear cell	124 (84.9)
Non-CCRCC	22 (15.1)
*Nuclear grade, no. (%)*	
I–II	61 (41.7)
III–IV	85 (58.3)
lymph node metastasis, no. (%)	9 (5.9)
Necrosis, no. (%)	66 (45.8)
*SSIGN score*	
Low-risk (score 2–4)	49 (33.5)
Intermediate-risk (score 5–7)	76 (52.1)
High-risk (score 8–11)	21 (13.4)

*IQR* interquartile range, *BMI* body mass index, *ASA* American Society of Anesthesiologists, *CCRCC* clear cell renal cell carcinoma

**Fig. 2:**
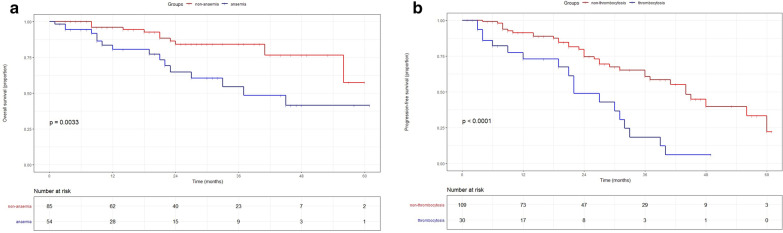
Kaplan-Meier curves showing the cumulative survival of patients with renal cell carcinoma and tumour thrombus categorized by anaemia and thrombocytosis. **a **The overall survival curve for anaemic and non-anaemic patients; **b** the progression-free survival curve for thrombocytosis and non-thrombocytosis patients

The baseline preoperative blood parameters of the study cohort are shown in Table 2. Univariate analysis revealed that anaemia, SSIGN score, and lymph node metastasis were substantial predictors of OS. Besides, thrombocytosis, anaemia, histologic subtype, nuclear grade, lymph node metastasis, SSIGN score were significant predictors of PFS (Table 3). Stepwise multivariable analysis revealed that anemia (HR 2.873, P = 0.008) and lymph node metastasis (HR 4.811, P = 0.015) were independent prognostic factors associated with OS. Besides, thrombocytosis (HR 2.324, P = 0.011), histologic subtype (HR 2.835, P = 0.004), nuclear grade (HR 2.069, P = 0.033), lymph node metastasis (HR 5.739, P = 0.001) were independent predictors of PFS. Furthermore, anaemia and thrombocytosis were significant prognostic factors independent of the SSIGN scores to predict OS and PFS respectively (Table 4). Using the Kaplan–Meier survival analysis we found that patients with preoperative anaemia exhibited a significantly increased mortality risk than those without it. Besides, preoperative thrombocytosis was also significantly associated with an increased risk of earlier recurrence or metastasis than those without it (Fig. 2).

**Table 2:** Preoperative blood parameters of patients with renal cell carcinoma and tumour thrombus treated surgically

Blood parameters (unit)	Median (IQR)
NEU (× 10^9^/L)	4.31 (3.53–5.04)
LYM(× 10^9^/L)	1.29 (1–1.62)
PLT (× 10^9^/L)	235 (185.25–299)
HGB (g/L)	123 (111–139)
ALB (g/L)	40 (35.55–43.05)
SCR (umol/L)	89.5 (80–108.5)
ALT (u/L)	16 (11.75–26.25)
AST(u/L)	18 (15–24)
SCa (mmol/L)	2.28 (2.18–2.38)
ALP (u/L)	80 (65–101)
NLR	3.43 (2.39–4.49)
PLR	184.81 (118.89–273.57)
AST/ALT	1.13 (0.83–1.45)

*NEU *neutrophil count, *LYM* lymphocyte count, *PLT* platelet count, *HGB* hemoglobin, *ALB* albumin, *SCR* serum creatinine, *SCa* serum calcium, *ALP* alkaline phosphatase

**Table 3:** Univariate Cox regression analysis of prognostic factors correlated with overall survival and progression-free survival

Variable	Overall survival			Progression-free survival		
	HR	95 %CL	P-value	HR	95 %CL	P-value
Age*	0.980	0.943–1.018	0.289	0.976	0.950–1.003	0.086
*Sex (male vs. female)*						
Male	Ref			Ref		
Female	0.874	0.330–2.315	0.787	0.801	0.390–1.645	0.545
*ASA score*						
I + II	Ref			Ref		
III	1.511	0.518–4.412	0.450	1.110	0.471–2.613	0.812
Symptom at presentation	1.858	0.745–4.629	0.184	1.878	0.978–3.603	0.058
*Histologic subtype*						
CCRCC	Ref			Ref		
Non-CCRCC	2.279	0.909–5.716	0.079	3.007	1.620–5.845	0.001
*Tumour thrombus level*						
0–II	Ref			Ref		
III–IV	1.259	0.525–3.022	0.606	1.149	0.609–2.167	0.669
Tumour size^*^	1.032	0.910–1.170	0.626	1.015	0.926–1.113	0.749
Invasion of venous wall	1.455	0.681–3.112	0.333	1.450	0.824–2.551	0.198
*Nuclear grade*						
I–II	Ref			Ref		
III–IV	2.283	0.955–5.456	0.063	2.368	1.255–4.468	0.008
Lymph node metastasis	5.622	1.872–16.888	0.002	3.871	1.504–9.962	0.005
Necrosis	1.889	0.850–4.198	0.119	1.709	0.946–3.028	0.066
*SSIGN score*						
Low-risk (score 2–4)	Ref			Ref		
Intermediate-risk (score 5–7)	2.158	0.771–6.039	0.143	2.311	1.125–4.749	0.023
High-risk (score 8–11)	5.653	1.808–17.677	0.003	3.799	1.598–9.033	0.003
*Blood parameters*						
NLR^*^	1.090	0.959–1.239	0.189	1.019	0.905–1.148	0.750
PLR^*^	1.001	0.998–1.004	0.377	1.002	1.000–1.004	0.084
AST/ALT^*^	1.117	0.553–2.256	0.758	1.212	0.983–1.765	0.238
Thrombocytosis	2.288	0.946–5.532	0.066	3.307	1.819–6.009	< 0.001
Anaemia	2.991	1.384–6.467	0.005	2.065	1.196–3.566	0.009
ALB^*^	0.959	0.902–1.021	0.191	0.979	0.935–1.026	0.384
SCR^*^	1.003	0.987–1.019	0.714	0.994	0.981–1.007	0.376
SCa^*^	0.666	0.064–6.885	0.733	3.740	0.769–18.205	0.102
ALP^*^	1.006	0.998–1.013	0.150	1.001	0.995–1.008	0.638

*As continuous variable

**Table 4:** Multivariate Cox regression analysis of blood parameter with clinicopathologic characteristics and SSIGN prognostic model

Variable	Overall survival			Progression-free survival		
	HR	95 %CL	P	HR	95 %CL	P
*Model 1*						
Age^1,2^	/	/	NS	/	/	NS
Symptom at presentation^1,2^	/	/	NS	/	/	NS
*Histologic subtype*^1,2^						
CCRCC	Ref			Ref		
Non-CCRCC	/	/	NS	2.835	1.403–5.728	0.004
*Nuclear grade*^1,2^						
I–II	Ref			Ref		
III–IV	/	/	NS	2.069	1.059–4.039	0.033
Lymph node metastasis^1,2^	4.811	1.365–16.961	0.015	5.739	2.136–15.420	0.001
Necrosis^2^	/	/	/	/	/	NS
*Blood parameters*						
PLR^2^	/	/	/	/	/	NS
Thrombocytosis^1,2^	/	/	NS	2.324	1.215–4.446	0.011
Anaemia^1,2^	2.873	1.310–6.299	0.008	/	/	NS
*Model 2*						
*SSIGN score*						
Low-risk (score 2–4)	Ref			Ref		
Intermediate-risk (score 5–7)	1.447	0.499–4.193	0.496	1.833	0.871–3.858	0.111
High-risk (score 8–11)	5.847	1.838–18.603	0.003	3.025	1.230–7.436	0.016
*Blood parameters*						
PLR^2^	/	/	/	/	/	NS
Thrombocytosis^1,2^	/	/	NS	2.375	1.245–4.469	0.008
Anaemia^1,2^	3.453	1.495–7.978	0.004	/	/	NS

*NS* not statistically significant

^1^The variable that was selected into the multivariate model correlated with overall survival

^2^The variable that was selected into the multivariate model correlated with progression-free survival

**Fig. 3:**
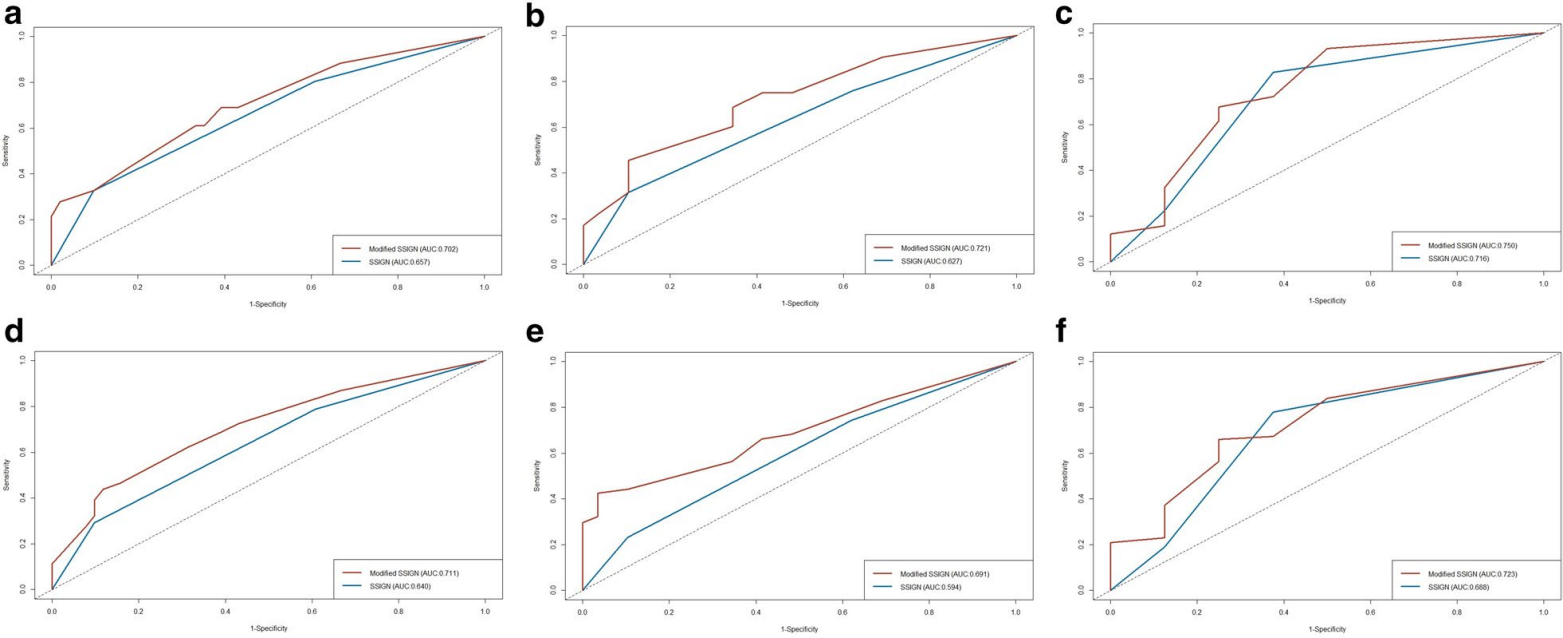
Time-dependent ROC curves for modified SSIGN score and SSIGN score relate to overall survival (OS) and progression-free survival (PFS). **a** 2-year OS; **b** 3-year OS; **c** 4-year OS; **d** 2-year PFS; **e** 3-year PFS; **f** 4-year PFS

To investigate the improvement in predictive ability by adding anaemia and thrombocytosis to SSIGN score, we performed time-dependent ROC curves and compared the AUCs of the modified SSIGN model to the conventional SSIGN model. Figure 3 illustrates the modified SSIGN score exhibited better predictive accuracy than the conventional SSIGN in predicting 2-year (AUCs: 702 vs. 0.657), 3-year (AUCs: 721 vs. 0.627), and 4-year (AUCs: 0.750 vs. 0.716) OS. Likewise, the modified SSIGN score exhibited better predictive accuracy than the conventional SSIGN in predicting 2-year (AUCs: 0.711 vs. 0.640), 3-year (AUCs: 0.691 vs. 0.594), and 4-year (AUCs: 0.723 vs. 0.688) PFS.

Furthermore, the association between preoperative anaemia and thrombocytosis with clinicopathological characteristics is shown in Table 5. Preoperative anaemia was related to more symptom at presentation (OR 3.348, P = 0.006), longer surgical time (OR 1.005, P = 0.001), more blood loss (OR 1.000, P = 0.018), more transfusion (OR:2.734, P = 0.004), higher thrombus level (OR 4.750, P = 0.001) and higher nuclear grade (OR 3.449, P = 0.001). Moreover, patients with thrombocytosis were associated with more symptoms at presentation (OR 7.784, P = 0.007).

**Table 5:** Odds ratios and 95% confidence interval for the association of anaemia and thrombocytosis with clinicopathological characteristics

Variable	Anaemia			Thrombocytosis		
	OR	95 %CL	P-value	OR	95 %CL	P-value
Age, yr	0.998	0.966–1.031	0.893	1.008	0.970–1.047	0.679
ASA score (III vs. I–II)	1.791	0.648–4.952	0.262	0.396	0.086–1.826	0.235
Symptom at presentation	3.348	1.405–7.977	0.006	7.784	1.767–34.298	0.007
Surgical time, min	1.005	1.002–1.008	0.001	1.000	0.997–1.003	0.880
Blood loss, ml	1.000	1.000–1.001	0.018	1.000	0.999–1.000	0.137
Transfusion	2.734	1.374–5.441	0.004	1.284	0.593–2.782	0.526
Segmental resection of vena cava	0.465	0.199–1.084	0.076	0.857	0.327–2.244	0.754
Tumour thrombosis level (III–IV vs. 0–II)	4.750	1.910–11.814	0.001	1.474	0.579–3.750	0.416
Tumour size, cm	0.988	0.883–1.107	0.840	1.135	0.996–1.294	0.057
Invasion of venous wall	1.381	0.670–2.846	0.382	0.973	0.409–2.311	0.950
Histologic subtype (non-CCRCC vs. CCRCC)	2.449	0.971–6.175	0.058	1.677	0.621–4.527	0.308
Nuclear grade (III–IV vs. I–II)	3.449	1.653–7.196	0.001	2.315	0.990–5.412	0.053
lymph node metastasis	0.884	0.203–3.850	0.869	0.446	0.053–3.758	0.458
Necrosis	1.488	0.762–2.908	0.245	1.245	0.576–2.689	0.577

## Discussion

In this study, from a high-volume centre, we examined the data related to 146 RCC and TT patients who underwent surgical treatment. We found that patients with preoperative anaemia had significantly adverse OS than those without it. Likewise, thrombocytosis was linked to poor PFS. In multivariable analysis, we found that preoperative anaemia and thrombocytosis were the independent worse prognostic factors even after adjusting for the other known pathologic prognostic factors and SSIGN scores. Furthermore, adding anaemia and thrombocytosis to the SSIGN score improved its predictive accuracy related to OS and PFS. To the best of our knowledge, this is the first study analysing preoperative blood parameters that specifically focused on the RCC population with TT.

Previously, anaemia has been suggested as a prognostic factor in RCC. Kim et al. retrospectively analysed data of 4260 patients that had non-metastatic RCC and found anaemia was highly associated with worse recurrence-free survival (RFS), cancer-specific survival (CSS), and overall survival (OS) [17]. *Jiwei Huang et al. *analysed the data of 352 papillary RCC patients and found that patients with preoperative anaemia had significantly worse RFS, CSS, and OS than those without it (P < 0.001). Multivariable analysis revealed that anaemia was an independent prognostic factor in terms of RFS, CSS, and OS (P < 0.001) [18]. Xia et al*.* conducted a meta-analysis and found preoperative anaemia was associated with increased all-cause mortality, cancer-specific mortality, and disease recurrence [19]. Our study found that preoperative anaemia was a predictor of worse OS and PFS. However, after controlling for pathologic features, preoperative anaemia was only independently associated with OS but not with PFS. To our knowledge, preoperative anaemia represented worse nutrition status of patients either due to long term poor consumption or blood loss, thus it could independently affect OS. In our study, anaemia exhibited a higher level of TT, higher nuclear grade, and more symptoms at presentation. We speculate that it is advanced tumour biology rather than anaemia that had a more significant impact on PFS, although this hypothesis needs to be verified externally.

Interestingly, thrombocytosis had been established as a worse prognostic predictor in various cancers, including nasopharyngeal carcinoma [20], gynecologic malignancies [21], melanoma [22], and colorectal cancer [23]. In RCC too, thrombocytosis could be regarded as a potential prognostic indicator. Several studies have shown that patients with preoperative thrombocytosis had worse survival than those without it [24–26]. Although none of the studies linked thrombocytosis to worse prognosis in RCC population with TT, a study found that bland thrombus in RCC with TT is linked to adverse survival [27]. Data from our study suggest that patients with thrombocytosis had an increased risk of recurrence or metastasis than those without it. Also, we found that thrombocytosis is an independent prognostic factor of PFS. Notably, the previous notion that platelets were mainly involved in limiting blood loss and promoting wound healing has been changed with recent preclinical and clinical findings. Studies now show that platelets can also promote tumorigenesis and metastasis through a crosstalk with cancer cells[28]. This rationalize preoperative thrombocytosis link to post-surgery higher risk of recurrence or metastasis. Therefore, we suggest that in cases of preoperative thrombocytosis, a follow-up plan should be closely monitored in populations with RCC and TT.

Nowadays many established prognostic models have been established to predict prognosis in patients with RCC [3]. Several clinicopathological features such as symptom at presentation, TNM stage, Fuhrman grade, and necrosis were regarded as prognostic factors in the current model [15]. To our knowledge, anaemia and thrombocytosis have already been added to the IMDC model to predict prognosis in patients with metastatic RCC [29]. Concerning nonmetastatic RCC, none of the models have included blood parameters. Since our study found that preoperative anaemia and thrombocytosis could serve as potential biomarkers predicting prognosis in patients with RCC and TT, we added these two factors to the SSIGN score and improved its predictive accuracy related to 2-year, 3-year, and 4-year OS and PFS. Thus, we propose that both anaemia and thrombocytosis should not be ignored as prognostic factors of worse survival in these patients.

Several factors such as NLR, PLR, AST/ALT, and ALB have shown their prognostic value in local and metastatic RCC [8–10, 30]. However, these blood parameters were not associated with prognosis in our study cohort. Previous studies demonstrated that these parameters reflected personal inflammatory conditions. To our knowledge, in patients with RCC and TT, the tumour thrombus was directly in contact with circulating blood. Thus, we speculated that the inflammatory condition was more complex in these unique populations and could not thoroughly be revealed by these inflammatory indices. Notably, Peyton et al. [9] also found that in patients with metastatic RCC and TT, NLR > 4 was associated with worse survival. We think that an insufficient number of patients included in our study could be another reason to not find any statistical differences in these factors.

Nevertheless, this retrospective study had several limitations. Firstly, as mentioned above, the insufficient number of patients, short follow-up time, and insufficient outcome events could have limited the study of several blood parameters. Therefore, future studies are needed to externally validate the proposed risk factors in a large prospective cohort. Secondly, we did not have access to data related to concomitant drugs, which could have influenced blood counts (e.g., steroids). Thirdly, our research is a single-institutional retrospective review, which inherently had missing data and confounding bias that were beyond our control. Despite these limitations, these findings are significant as this is the only study to date that specifically focused on the impact of preoperative blood parameters in RCC patients with TT.

## Conclusions

Both preoperative anaemia and thrombocytosis are the important independent prognostic factors in RCC patients with TT. We found that anaemia is associated with an increased risk of mortality while thrombocytosis is linked to a higher risk of earlier recurrence or metastasis. Furthermore, both preoperative anaemia and thrombocytosis could be clinically useful for risk stratifying patients undergoing surgery for non-metastatic RCC with TT.

## Data Availability

The datasets used and/or analysed during the current study are available.

## Abbreviations

RCC: Renal cell carcinoma
TT: Tumour thrombus
SCR: Serum creatinine
ALB: Albumin
HGB: Haemoglobin
NLR: Neutrophil-lymphocyte ratio
PLR: Platelet lymphocyte ratio
NEU: Neutrophil count
PLT: Platelet count
LYM: Lymphocyte count
SCa: Serum calcium
ALP: Alkaline phosphatase
BMI: Body mass index
ASA: American Society of Anesthesiologists score
OS: Overall survival
PFS: Progression-free survival

## References

[1] Ferlay J, Colombet M, Soerjomataram I, Dyba T, Randi G, Bettio M, Gavin A, Visser O, Bray F (2018). Cancer incidence and mortality patterns in Europe: estimates for 40 countries and 25 major cancers in 2018. Eur J Cancer.

[2] Keegan KA, Schupp CW, Chamie K, Hellenthal NJ, Evans CP, Koppie TM (2012). Histopathology of surgically treated renal cell carcinoma: survival differences by subtype and stage. J Urol.

[3] Sun M, Shariat SF, Cheng C, Ficarra V, Murai M, Oudard S, Pantuck AJ, Zigeuner R, Karakiewicz PI (2011). Prognostic factors and predictive models in renal cell carcinoma: a contemporary review. Eur Urol.

[4] Greten FR, Grivennikov SI (2019). Inflammation and cancer: triggers, mechanisms, and consequences. Immunity.

[5] Bahig H, Taussky D, Delouya G, Nadiri A, Gagnon-Jacques A, Bodson-Clermont P, Soulieres D (2015). Neutrophil count is associated with survival in localized prostate cancer. BMC Cancer.

[6] Zhang J, Zhou X, Ding H, Wang L, Liu S, Liu Y, Chen Z (2020). The prognostic value of routine preoperative blood parameters in muscle-invasive bladder cancer. BMC Urol.

[7] Huszno J, Kolosza Z, Mrochem-Kwarciak J, Rutkowski T, Skladowski K (2019). The role of neutrophil-lymphocyte ratio, platelet-lymphocyte ratio, and platelets in the prognosis of metastatic renal cell carcinoma. Oncology.

[8] Hu H, Yao X, Xie X, Wu X, Zheng C, Xia W, Ma S (2017). Prognostic value of preoperative NLR, dNLR, PLR and CRP in surgical renal cell carcinoma patients. World J Urol.

[9] Peyton CC, Abel EJ, Chipollini J, Boulware DC, Azizi M, Karam JA, Margulis V, Master VA, Matin SF, Raman JD (2020). The value of neutrophil to lymphocyte ratio in patients undergoing cytoreductive nephrectomy with thrombectomy. Eur Urol Focus.

[10] Bezan A, Mrsic E, Krieger D, Stojakovic T, Pummer K, Zigeuner R, Hutterer GC, Pichler M (2015). The preoperative AST/ALT (De Ritis) ratio represents a poor prognostic factor in a cohort of patients with nonmetastatic renal cell carcinoma. J Urol.

[11] Chrom P, Stec R, Bodnar L, Szczylik C (2018). Incorporating neutrophil-to-lymphocyte ratio and platelet-to-lymphocyte ratio in place of neutrophil count and platelet count improves prognostic accuracy of the international metastatic renal cell carcinoma database consortium model. Cancer Res Treat.

[12] Lardas M, Stewart F, Scrimgeour D, Hofmann F, Marconi L, Dabestani S, Bex A, Volpe A, Canfield SE, Staehler M (2016). Systematic review of surgical management of nonmetastatic renal cell carcinoma with vena caval thrombus. Eur Urol.

[13] Blute ML, Leibovich BC, Lohse CM, Cheville JC, Zincke H (2004). The Mayo Clinic experience with surgical management, complications and outcome for patients with renal cell carcinoma and venous tumour thrombus. BJU Int.

[14] Moch H, Cubilla AL, Humphrey PA, Reuter VE, Ulbright TM (2016). The 2016 WHO classification of tumours of the urinary system and male genital organs-part A: renal, penile, and testicular tumours. Eur Urol.

[15] Frank I, Blute ML, Cheville JC, Lohse CM, Weaver AL, Zincke H (2002). An outcome prediction model for patients with clear cell renal cell carcinoma treated with radical nephrectomy based on tumor stage, size, grade and necrosis: the SSIGN score. J Urol.

[16] Heagerty PJ, Lumley T, Pepe MS (2000). Time-dependent ROC curves for censored survival data and a diagnostic marker. Biometrics.

[17] Kim SH, Park B, Hwang EC, Hong SH, Jeong CW, Kwak C, Byun SS, Chung J (2019). Retrospective multicenter long-term follow-up analysis of prognostic risk factors for recurrence-free, metastasis-free, cancer-specific, and overall survival after curative nephrectomy in non-metastatic renal cell carcinoma. Front Oncol.

[18] Huang J, Feldman AS, Dong L, Cornejo K, Liu Q, Dahl DM, Wu S, Blute ML, Huang Y, Wu CL (2015). Preoperative anemia as an independent prognostic indicator of papillary renal cell carcinoma. Clin Genitourin Cancer.

[19] Xia L, Guzzo TJ (2017). Prognostic significance of preoperative anemia in patients undergoing surgery for renal cell carcinoma: a meta-analysis. Anticancer Res.

[20] Chen YP, Zhao BC, Chen C, Shen LJ, Gao J, Mai ZY, Chen MK, Chen G, Yan F, Liu S (2015). Pretreatment platelet count improves the prognostic performance of the TNM staging system and aids in planning therapeutic regimens for nasopharyngeal carcinoma: a single-institutional study of 2,626 patients. Chin J Cancer.

[21] Menczer J (2017). Preoperative elevated platelet count and thrombocytosis in gynecologic malignancies. Arch Gynecol Obstet.

[22] Rachidi S, Md P, Kaur MM, Lautenschlaeger TM, Li Z, Md P (2019). Platelet count correlates with stage and predicts survival in melanoma. Platelets.

[23] Zhu X, Cao Y, Lu P, Kang Y, Lin Z, Hao T, Song Y (2018). Evaluation of platelet indices as diagnostic biomarkers for colorectal cancer. Sci Rep.

[24] Życzkowski M, Prokopowicz G, Taborowski P, Nowakowski K, Rajwa P, Stelmach P, Paradysz A (2018). Basic parameters of blood count, serum sodium, and creatinine as prognostic factors for renal cell carcinoma at five-year follow-up. Med Sci Monit Int Med J Exp Clin Res.

[25] Choi JY, Ko YH, Song PH (2016). Clinical significance of preoperative thrombocytosis in patients who underwent radical nephrectomy for nonmetastatic renal cell carcinoma. Investig Clin Urol.

[26] Hutterer GC, Krieger D, Mrsic E, Pohlmann K, Bezan A, Stojakovic T, Pummer K, Zigeuner R, Pichler M (2015). Preoperative leucocytosis, thrombocytosis and anemia as potential prognostic factors in non-metastatic renal cell carcinoma. Anticancer Res.

[27] Hutchinson R, Rew C, Chen G, Woldu S, Krabbe LM, Meissner M, Sheth K, Singla N, Shakir N, Master VA (2018). The adverse survival implications of bland thrombus in renal cell carcinoma with venous tumor thrombus. Urology.

[28] Haemmerle M, Stone RL, Menter DG, Afshar-Kharghan V, Sood AK (2018). The platelet lifeline to cancer: challenges and opportunities. Cancer Cell.

[29] Heng DY, Xie W, Regan MM, Harshman LC, Bjarnason GA, Vaishampayan UN, Mackenzie M, Wood L, Donskov F, Tan MH (2013). External validation and comparison with other models of the International Metastatic Renal-Cell Carcinoma Database Consortium prognostic model: a population-based study. Lancet Oncol.

[30] Peng D, Zhang CJ, Tang Q, Zhang L, Yang KW, Yu XT, Gong Y, Li XS, He ZS, Zhou LQ (2018). Prognostic significance of the combination of preoperative hemoglobin and albumin levels and lymphocyte and platelet counts (HALP) in patients with renal cell carcinoma after nephrectomy. BMC Urol.

